# Transmission of Mental Disorders in Adolescent Peer Networks

**DOI:** 10.1001/jamapsychiatry.2024.1126

**Published:** 2024-05-22

**Authors:** Jussi Alho, Mai Gutvilig, Ripsa Niemi, Kaisla Komulainen, Petri Böckerman, Roger T. Webb, Marko Elovainio, Christian Hakulinen

**Affiliations:** 1 Department of Psychology, University of Helsinki, Helsinki, Finland; 2 School of Business and Economics, University of Jyväskylä, Jyväskylä, Finland; 3 Centre for Mental Health and Safety, Division of Psychology & Mental Health, University of Manchester, Manchester Academic Health Sciences Centre, Manchester, United Kingdom; 4 National Institute for Health and Care Research Greater Manchester Patient Safety Research Collaboration, Manchester, United Kingdom; 5 Finnish Institute for Health and Welfare, Helsinki, Finland

## Abstract

**Importance:**

Previous research indicates that mental disorders may be transmitted from one individual to another within social networks. However, there is a lack of population-based epidemiologic evidence that pertains to the full range of mental disorders.

**Objective:**

To examine whether having classmates with a mental disorder diagnosis in the ninth grade of comprehensive school is associated with later risk of being diagnosed with a mental disorder.

**Design, Setting, and Participants:**

In a population-based registry study, data on all Finnish citizens born between January 1, 1985, and December 31, 1997, whose demographic, health, and school information were linked from nationwide registers were included. Cohort members were followed up from August 1 in the year they completed ninth grade (approximately aged 16 years) until a diagnosis of mental disorder, emigration, death, or December 31, 2019, whichever occurred first. Data analysis was performed from May 15, 2023, to February 8, 2024.

**Exposure:**

The exposure was 1 or more individuals diagnosed with a mental disorder in the same school class in the ninth grade.

**Main Outcomes and Measures:**

Being diagnosed with a mental disorder during follow-up.

**Results:**

Among the 713 809 cohort members (median age at the start of follow-up, 16.1 [IQR, 15.9-16.4] years; 50.4% were males), 47 433 had a mental disorder diagnosis by the ninth grade. Of the remaining 666 376 cohort members, 167 227 persons (25.1%) received a mental disorder diagnosis during follow-up (7.3 million person-years). A dose-response association was found, with no significant increase in later risk of 1 diagnosed classmate (HR, 1.01; 95% CI, 1.00-1.02), but a 5% increase with more than 1 diagnosed classmate (HR, 1.05; 95% CI, 1.04-1.06). The risk was not proportional over time but was highest during the first year of follow-up, showing a 9% increase for 1 diagnosed classmate (HR, 1.09; 95% CI, 1.04-1.14), and an 18% increase for more than 1 diagnosed classmate (HR, 1.18; 95% CI, 1.13-1.24). Of the examined mental disorders, the risk was greatest for mood, anxiety, and eating disorders. Increased risk was observed after adjusting for an array of parental, school-level, and area-level confounders.

**Conclusions and Relevance:**

The findings of this study suggest that mental disorders might be transmitted within adolescent peer networks. More research is required to elucidate the mechanisms underlying the possible transmission of mental disorders.

## Key Points

**Question:**

Is having peers with a mental disorder in the same social network during adolescence associated with later risk of mental disorder?

**Findings:**

In this cohort study including more than 700 000 individuals in Finland, analysis of nationwide, interlinked registry data found that having classmates diagnosed with a mental disorder in the ninth grade of comprehensive school was associated with increased risk of receiving a mental disorder diagnosis later in life. Increased risk remained after adjusting for an array of parental, school-level, and area-level confounders.

**Meaning:**

The findings of this study suggest that mental disorders might be socially transmitted within adolescent peer networks.

## Teaser

This cohort study examines the transmission of mental disorders among adolescents observed in follow-up after school exposure to 1 or more classmates diagnosed with a mental disorder.

## Introduction

Mental disorders are major contributors to the global disease burden, having detrimental individual, societal, and economic impacts.^1,2,3^ When investigating the impacts of mental disorders, the focus has typically been on the directly affected individual. It is, however, well established that the immediate family members are also adversely affected.^4^ Empirical findings suggest that harmful effects extend beyond the immediate family to friends and peers via social networks.^5,6,7,8^ For example, a longitudinal cohort study where a social network of 12 067 adults was followed up over 20 years indicated that depressive symptoms appear to transmit from person to person.^7^

Investigating the transmission of mental disorders is especially important in childhood and adolescence. These are key developmental periods when the onset of many mental disorders is most likely to occur^9^ and when enduring peer networks and behaviors are established,^10^ particularly in the context of peer relationships.^11,12^ Understanding the role of peer effects in early-life mental health problems would also offer tools for more successful prevention and intervention measures, thus reducing the economic and societal burden of mental disorders. Yet, despite a few survey studies reporting that adolescents may experience increased mental health symptoms when exposed to friends or peers with mental health problems,^6,13^ large-scale studies on the potential peer influences of mental disorders in youth are lacking.

When analyzing network associations, an additional difficulty arises from people’s tendency to network with others who have similar traits. Such self-selection bias (or homophily) can be mitigated by using institutionally imposed networks, such as school classes, which are not formed endogenously by the pupils choosing similar others as classmates. In Finland, parents also cannot directly choose their children’s comprehensive school; instead, the school is selected based on the proximity to the residential location. Moreover, school class constitutes arguably the most substantial peer network in childhood and adolescence due to the amount of time spent together with classmates.^14,15,16^ In the present study, we combined the use of registry data and institutionally imposed peer networks to study the possible transmission of mental disorders among peers. More specifically, we used nationwide, interlinked Finnish registers to examine whether mental disorders are transmitted within peer networks formed by adolescents who were in the same class in the ninth grade of comprehensive school.

## Methods

### Study Population

The study population comprised all Finnish citizens born between January 1, 1985, and December 31, 1997, whose demographic, health, and school information was linked from several nationwide registers based on unique identification numbers, assigned to all Finnish residents since 1969. Death or emigration before the start of follow-up, move to the municipality of the school later than 3 years before the start of follow-up, and, for those who were born outside Finland, immigration to Finland after school starting age (ie, August 1 in the year they turn 7 years) were used as exclusion criteria. The ethics committee of the Finnish Institute for Health and Welfare approved the study plan (THL/184/6.02.01/2023§933). Data were linked with the permission of Statistics Finland (TK-53-1696-16) and the Finnish Institute for Health and Welfare. According to Finnish law, informed consent is not required from participants in register-based studies. This study followed the Strengthening the Reporting of Observational Studies in Epidemiology (STROBE) reporting guideline.

The school information was based on the National Joint Application Register. It discloses school class divisions in the final year (ie, ninth grade) of comprehensive school. Individuals with missing or insufficient school class information were excluded. To exclude the smallest classes and omit incorrect registry information (eg, implausibly large classes), individuals in classes with fewer than 10 or more than 40 pupils were excluded. Of the remaining 713 809 individuals, 47 433 had a mental disorder diagnosis (*International Statistical Classification of Diseases and Related Health Problems, Tenth Revision* [*ICD-10*] diagnoses F10-F50 or F90-F98) before follow-up commenced and were therefore excluded from follow-up. The remaining 666 376 individuals in 860 schools and 39 992 classes (median, 6 [IQR, 4-7] classes per grade) formed the outcome population and were followed up from August 1 in the year during which they completed ninth grade (approximately aged 16 years) until the first diagnosed mental disorder, death, emigration, or end of follow-up on December 31, 2019, whichever occurred first. The maximum length of follow-up was thus from August 1, 2001, to December 31, 2019. For the annual number of mental disorder diagnoses and proportion of exposed classes and individuals in the outcome population, see eTable 1 in Supplement 1.

### Mental Disorders

Information on mental disorders was acquired from the Care Register for Health Care of the Finnish Institute for Health and Welfare. It contains information on all inpatient hospital admissions in Finland since 1970, hospital outpatient care since 1998, and primary care since 2011. Mental disorders were diagnosed according to the *International Statistical Classification of Diseases Spectrum Health Problems, Eighth Revision*, from 1970 to 1986; *International Classification of Diseases, Ninth Revision*, from 1987 to 1995; and *ICD-10* since 1996.

For the study population, we used the following mental disorder diagnosis categories: substance misuse disorders (F10-F19), schizophrenia spectrum disorders (F20-F29), mood disorders (F30-F39), anxiety disorders (F40-F48), eating disorders (F50), and behavioral and emotional disorders (F90-F98). Additionally, categories of internalizing disorders (F30-F39, F40-F48, F93-F94) and externalizing disorders (F10-F19, F90-F92) were constructed.

### Covariates

We included the following demographic, socioeconomic, and intergenerational variables as covariates: sex (0 = male, 1 = female), birth year, degree of urbanicity in residential location (0 = unknown, 1 = urban, 2 = semiurban, 3 = rural) based on the urban-rural classification of the Finnish Environment Institute, morbidity index of the municipality by the Finnish Institute for Health and Welfare in quintiles (0 = 1st quintile, 1 = 2nd quintile, 2 = 3rd quintile, 3 = 4th quintile, 4 = 5th quintile; as data were not available for 2001, data from 2002 were used instead), proportion of people without upper secondary or higher educational levels in the municipality in quintiles (0 = 1st quintile, 1 = 2nd quintile, 2 = 3rd quintile, 3 = 4th quintile, 4 = 5th quintile), proportion of unemployed people in the municipality in quintiles (0 = 1st quintile, 1 = 2nd quintile, 2 = 3rd quintile, 3 = 4th quintile, 4 = 5th quintile), size of school class (number of pupils), size of school’s ninth grade (number of pupils), parental education level at time of child’s ninth grade (0 = comprehensive, 1 = upper secondary, 2 = higher education), parental income level in quintiles relative to study population at time of child’s ninth grade (0 = unknown, 1 = 1st quintile, 2 = 2nd quintile, 3 = 3rd quintile, 4 = 4th quintile, 5 = 5th quintile), and parental mental health history at the time of child’s ninth grade (0 = no mental disorder diagnosis, 1 = any mental disorder diagnosis). The median population of Finnish municipalities in 2001-2013 was 6530 inhabitants. Any mental disorder diagnosis (F00-F99) was used for parental mental health history.

### Statistical Analysis

Data analysis was conducted from May 15, 2023, to February 8, 2024. We used mixed-effects Cox proportional hazards regression models with a random intercept per school to estimate the association between having a classmate with a mental disorder diagnosis and later risk of being diagnosed with a mental disorder. Random intercept per school was included to account for the varying predisposition to mental health problems between schools. Results are reported as hazard ratios (HRs). Schoenfeld residuals were calculated to test the proportional hazards assumption of Cox regression models and estimate the time dependence of the HR throughout the entire follow-up period. We also separately estimated the HRs in shorter intervals: first year of follow-up, years 2 and 3, years 4 and 5, and after year 5. In the primary analyses, all models were adjusted for sex, birth year, area-level urbanicity, area-level morbidity, area-level educational level, area-level employment rate, school class size, school’s ninth grade size, parental educational level, parental income, and parental mental health, and included a 3-level exposure variable for diagnosed classmates (0 = none, 1 = 1, 2 = >1).

As a sensitivity analysis, we estimated the Cox proportional hazards regression models separately for all the mental disorder diagnosis categories (with the same category both as exposure and outcome). Since the number of cases where more than 1 diagnosed classmate was low for some diagnosis categories, a binary variable indicating the presence or lack of individuals diagnosed in the class (0 = no, 1 = yes) was used as a secondary exposure. As additional sensitivity analyses, we limited exposure diagnoses to 3 years preceding the start of follow-up, specifically focusing on diagnoses received during lower secondary education (grades 7-9) and, to control for incorrect registry information regarding school class divisions (eg, implausibly large classes), only considered school classes with sizes falling within 5th and 95th percentiles (corresponding to classes with 12-25 pupils). We also assessed the attenuating impact of the covariates and random intercepts by adding a random intercept per school and covariates in 3 domains (parental, school-level, and area-level) separately in a crude model adjusted for sex and birth year. Moreover, to elucidate possible differences in the 2001-2013 study period, we stratified it into 3 shorter time periods: 2001-2004, 2005-2008, and 2009-2013. A 2-tailed *P* value <.05 was considered to indicate statistical significance. The statistical analyses were done using Stata, version 16.1 (StataCorp LLC) and R Statistical Software, version 4.2.2 (R Foundation for Statistical Computing) survival (version 3.4.0) and coxme (version 2.2.18.1) packages.

## Results

### Incidence and HRs

Among the 713 809 cohort members, 50.4% were male and 49.6% were female. Median age at the start of follow-up was 16.1 (IQR, 15.9-16.4) years. Descriptive statistics of the exposure and outcome populations are reported in Table 1 (additional descriptive statistics are provided in eTable 2 in Supplement 1). During 7.3 million person-years of follow-up time, with a median of 11.4 (IQR, 7.4-14.4) years, 167 227 cohort members (25.1%) were diagnosed with a mental disorder, corresponding to an incidence rate of 2283 per 100 000 person-years at risk. Table 2 reports the incidence rates and HRs for the association between classmates diagnosed with a mental disorder and later risk of being diagnosed with a mental disorder for each diagnosis category. Having more than 1 diagnosed classmate with any of the examined mental disorders was associated with a 5% higher risk of later diagnosis (HR, 1.05; 95% CI, 1.04-1.06). The SD of the random intercepts for schools was 0.12, indicating that pupils in a school that was 1 SD above the mean had (e^0.12^ = 1.13) 13% higher risk of being diagnosed with a mental disorder. Diagnosis-specific analyses revealed positive associations for mood, anxiety, and eating disorders, as well as the internalizing disorders category, even with only 1 diagnosed classmate. For behavioral and emotional disorders, as well as the externalizing disorders category, the findings were significant only with more than 1 diagnosed classmate.

**Table 1.** Descriptive Profile of the Study Cohort’s Characteristics

Characteristic	No. (%)
Sex	
Male	360 041 (50.4)
Female	353 768 (49.6)
Birth year	
1985	55 610 (7.8)
1986	54 218 (7.6)
1987	53 401 (7.5)
1988	56 209 (7.9)
1989	56 062 (7.9)
1990	57 641 (8.1)
1991	56 738 (8.0)
1992	56 552 (7.9)
1993	56 152 (7.9)
1994	56 143 (7.9)
1995	54 890 (7.7)
1996	51 898 (7.3)
1997	48 295 (6.8)
Mental disorder diagnosed prior to follow-up, *ICD-10*	
F10-F19 Substance misuse disorders	3458 (0.5)
F20-F29 Schizophrenia spectrum disorders	1142 (0.2)
F30-F39 Mood disorders	12 472 (1.7)
F40-F48 Anxiety disorders	13 730 (1.9)
F50 Eating disorders	4012 (0.6)
F90-F98 Behavioral and emotional disorders	26 532 (3.7)
Internalizing disorders	26 303 (3.7)
Externalizing disorders	14 152 (2.0)
Any of the above	47 433 (6.6)

Abbreviation: *ICD-10*, *International Statistical Classification of Diseases and Related Health Problems, Tenth Revision*.

**Table 2.** Associations Between Having Ninth-Grade Classmates With a Mental Disorder Diagnosis and Later Risk of Being Diagnosed With a Mental Disorder^a^

Mental disorder^b^	No.	Incidence rate	Diagnoses in class (exposure)						
			None, No. [reference]	1			>1		
				No.	HR (95% CI)	*P* value	No.	HR (95% CI)	*P* value
F10-F19 Substance misuse	30 939	382	28 510	2225	1.02 (0.97-1.06)	.42	204	1.07 (0.93-1.23)	.33
F20-F29 Schizophrenia spectrum	9924	121	9646	273	1.11 (0.99-1.26)	.08	5	1.02 (0.42-2.45)	.97
F30-F39 Mood	82 061	1050	61 845	16 183	1.04 (1.02-1.06)	<.001	4033	1.10 (1.07-1.14)	<.001
F40-F48 Anxiety	117 077	1522	86 401	24 476	1.03 (1.01-1.04)	<.001	6200	1.03 (1.00-1.06)	.03
F50 Eating	7333	89	6515	743	1.10 (1.02-1.19)	.01	75	1.29 (1.03-1.63)	.03
F90-F98 Behavioral/emotional	12 690	155	6963	3697	1.02 (0.98-1.07)	.26	2030	1.11 (1.05-1.17)	<.001
Internalizing	149 243	2001	84 562	43 426	1.03 (1.02-1.04)	<.001	21 255	1.06 (1.04-1.08)	<.001
Externalizing	34 851	431	25 552	7190	1.03 (1.00-1.05)	.05	2109	1.14 (1.09-1.20)	<.001
Any of the above	167 227	2283	62 314	53 860	1.01 (1.00-1.02)	.12	51 053	1.05 (1.04-1.06)	<.001

Abbreviation: HR, hazard ratio.

^a^ The Cox proportional hazards models were adjusted for sex, birth year, school class size, school’s ninth grade size, area-level urbanicity, area-level morbidity, area-level educational level, area-level employment rate, parental educational level, parental income, and parental mental health, with a random intercept per school.

^b^ *International Statistical Classification of Diseases and Related Health Problems, Tenth Revision* coding used.

### Time Dependence of the HRs

**Figure 1.**
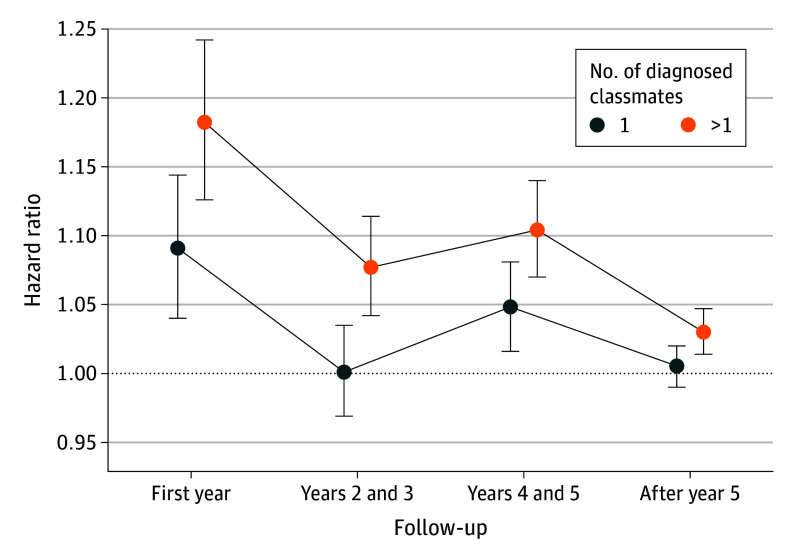
Associations Between Having Ninth-Grade Classmates With a Mental Disorder Diagnosis and Later Risk of Being Diagnosed With a Mental Disorder Hazard ratios with 95% CIs for the associations between having 1 or more than 1 ninth-grade classmate with any of the examined mental disorder diagnoses (*International Statistical Classification of Diseases and Related Health Problems, Tenth Revision,* codes F10-F50 or F90-F98) and later risk of being diagnosed with a mental disorder in 4 follow-up time windows. The Cox proportional hazards models were adjusted for sex, birth year, school class size, school’s ninth grade size, area-level urbanicity, area-level morbidity, area-level educational level, area-level employment rate, parental educational level, parental income, and parental mental health, with a random intercept per school. For the diagnosis-specific results with the 3-level exposure, see Figure 2; eFigure 2 in Supplement 1.

**Figure 2.**
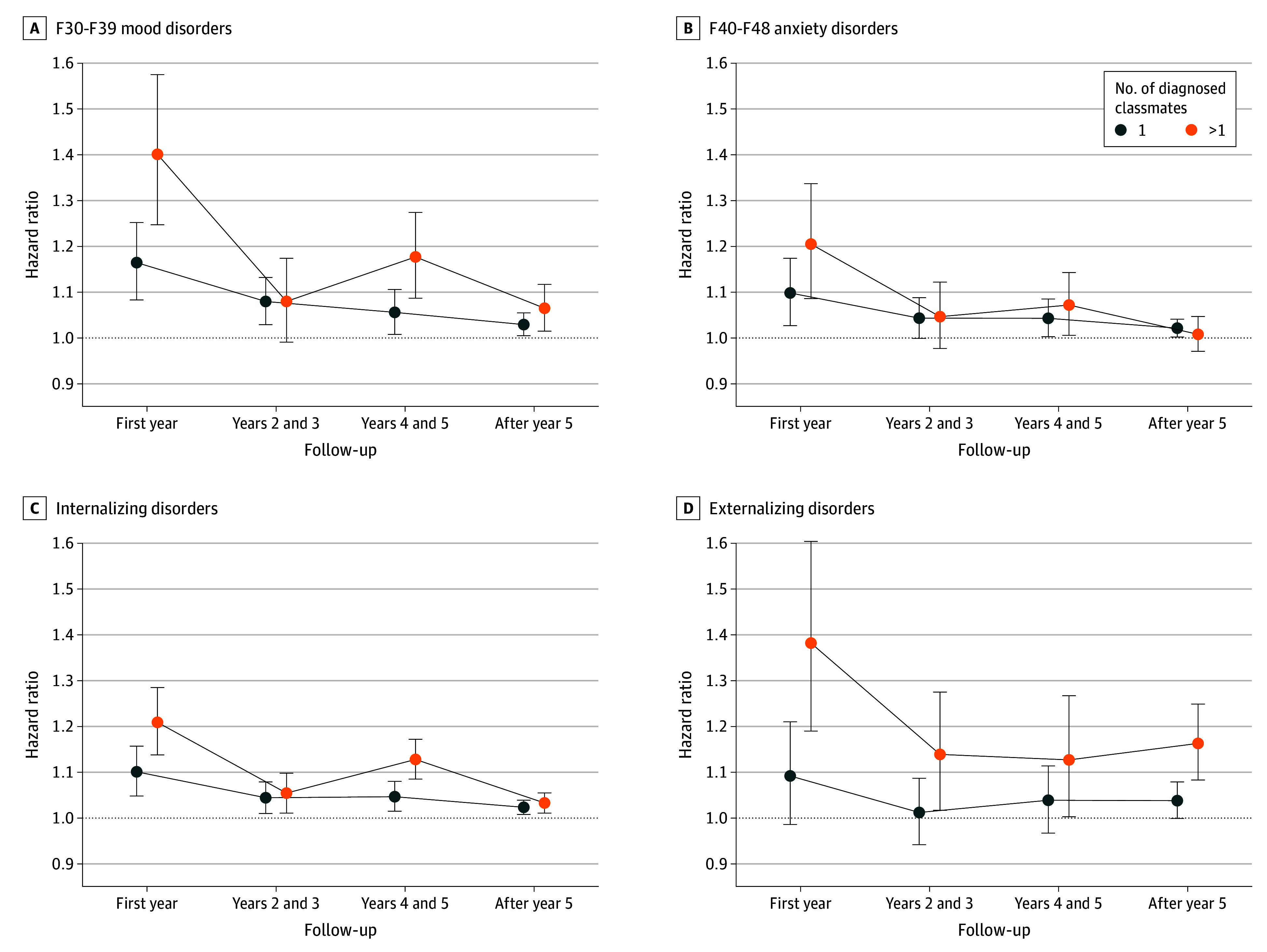
Diagnosis-Specific Associations Between Having Ninth-Grade Classmates With a Mental Disorder Diagnosis and Later Risk of Being Diagnosed With a Mental Disorder Hazard ratios with 95% CIs shown for mood (*International Statistical Classification of Diseases and Related Health Problems, Tenth Revision* [*ICD-10*], codes F30-F39) (A), anxiety (*ICD-10* codes F40-F48) (B), and internalizing (C) and externalizing (D) disorders in 4 follow-up time windows. The Cox proportional hazards models were adjusted for sex, birth year, school class size, school’s ninth grade size, area-level urbanicity, area-level morbidity, area-level educational level, area-level employment rate, parental educational level, parental income, and parental mental health, with a random intercept per school. For the diagnosis-specific associations for substance misuse, schizophrenia spectrum, eating, and behavioral and emotional disorders, see eFigure 2 in Supplement 1.

Schoenfeld residual-based tests showed that the proportional hazards assumption held only for the schizophrenia spectrum, eating, and behavioral and emotional disorders models. In contrast, models for other diagnosis categories displayed larger coefficient values (or HRs) earlier in follow-up (eFigure 1 in Supplement 1). Figure 1 shows the HRs for the association between diagnosed classmates and later risk of being diagnosed with any of the examined mental disorders in 4 shorter follow-up time windows (Figure 2; eFigure 2 and eTable 3 in Supplement 1 provide diagnosis-specific results). During the first year of follow-up, the risk of being diagnosed was 9% higher with 1 diagnosed classmate (HR, 1.09; 95% CI, 1.04-1.14) and 18% higher with more than 1 diagnosed classmate (HR, 1.18; 95% CI, 1.13-1.24). After the first year of follow-up, the risk of being diagnosed was statistically significant during years 4 and 5 with 1 diagnosed classmate with a mental disorder and in all 3 time windows with more than 1 diagnosed classmate with a mental disorder.

### Sensitivity Analyses

eFigure 3 in Supplement 1 shows the HRs for the association using binary exposure (see also eTable 4a in Supplement 1). The risk of being diagnosed with any mental disorder was 3% higher during the entire follow-up period (HR, 1.03; 95% CI, 1.02-1.04), 13% higher during the first year of follow-up (HR, 1.13; 95% CI, 1.08-1.18), and significantly increased also in the later time windows. Diagnosis-specific analyses showed that the risk was significantly increased for mood, anxiety, and internalizing disorders in each follow-up time window, with the greatest risks observed during the first year. For example, the risk of being diagnosed with a mood disorder was 21% higher during the first year of follow-up when a pupil was exposed to mood disorder (HR, 1.21; 95% CI, 1.13-1.29).

The results remained similar when considering only more recent exposure (diagnosis received during lower secondary education, ie, grades 7-9) as opposed to all previous childhood diagnoses (eTable 4b in Supplement 1), the most noticeable difference being that the association became statistically significant for schizophrenia spectrum disorders (HR, 1.17; 95% CI, 1.02-1.34). When repeating the analysis by limiting school class sizes within the 5th and 95th percentiles (12-25 pupils), the results remained similar, with no notable differences (eTable 4c in Supplement 1).

Assessing the impact of the different covariate domains, a crude model adjusted only for sex and birth year without a random intercept per school showed the highest HRs for all diagnosis categories. In contrast, a model including additional covariates describing area-level characteristics showed the largest reduction in HRs (eTable 5 in Supplement 1). For example, having classmates with a mood disorder diagnosis was associated with a 32% higher risk of being diagnosed with a mood disorder during the first year of follow-up (HR, 1.32; 95% CI, 1.23-1.41) in a model adjusted for sex and birth year without a random intercept per school. Including a random intercept per school decreased the risk to 24% (HR, 1.24; 95% CI, 1.16-1.32) and including covariates describing area-level characteristics further decreased the risk to 22% (HR, 1.22; 95% CI, 1.14-1.30). We also assessed differences in the 2001-2013 study period by stratifying it into 3 shorter periods (eTable 6 in Supplement 1). While the HRs showed a slight increase trend from the earliest to the latest period, the differences between the periods were statistically nonsignificant.

## Discussion

In our analysis of nationwide, interlinked registry data, including more than 700 000 individuals from 860 comprehensive schools in Finland, we found an association between having peers diagnosed with a mental disorder during adolescence and an increased risk of receiving a mental disorder diagnosis later in life. This risk was most pronounced in the first year of follow-up. The association showed a dose-response relationship, with higher risk when multiple diagnosed individuals were in the peer network. Of the mental disorders examined, the risk was greatest for mood, anxiety, and eating disorders. These associations were not explained by differences in area-level general morbidity or socioeconomic characteristics, parental mental disorders or socioeconomic position during childhood, or random differences in predisposition to mental health problems occurring among schools’ student populations.

To our knowledge, the present study is the largest and most comprehensive investigation on this topic to date. Our findings are consistent with previous studies reporting clustering of mood and/or anxiety symptoms in social networks of adolescents^6,13^ and adults,^5,7,8^ as well as with evidence suggesting similar social transmission of eating disorders.^17^ For example, a longitudinal survey study with a school-based design showed that exposure to peers with depressive symptoms in the same school grade was associated with more depressive symptoms in a sample of 8290 adolescents.^6^ Although using institutionally imposed peer network (eg, school grade or class) mitigates the self-selection bias often compromising studies on network peer effects, the study by Lee and Lee^6^ is not immune to biases related to selection and attrition that are typical in longitudinal survey studies. We sought to minimize these biases by using institutionally imposed peer networks (school classes) in combination with population-wide registry data and found that exposure to a peer with a mental disorder is associated with an increased risk of mental disorder across several different diagnosis categories.

If mental disorders are transmitted socially via peer networks, the phenomenon could be explained by several mechanisms. One plausible mechanism is the normalization of mental disorders through increased awareness and receptivity to diagnosis and treatment when having individuals with diagnosis in the same peer network.^18^ Similarly, having individuals with no diagnosis in the peer network might discourage seeking help for any underlying mental health problems. The observed higher risks of being diagnosed during the first year of follow-up after the exposure are consistent with this mechanism. Namely, due to diagnostic delay, the brief latency between exposure and diagnosis challenges the likelihood of harmful contagion occurring without an already existing, undiagnosed disorder. For some diagnosis categories, such as eating disorders, transmission could also occur through processes of peer social influence to which adolescents are particularly susceptible.^19^ Another possible mechanism facilitating the transmission of certain mental disorders, such as depression, pertains to direct interpersonal contagion. For instance, it is conceivable that long-term exposure to a depressive individual could lead to gradual development of depressive symptoms through the well-established neural mechanisms of emotional contagion.^20^

### Strengths and Limitations

The primary strengths of the present study are its use of a nationwide study population tracked from adolescence up to age 34 years, inclusion of interlinked primary and secondary health care registry data, and reliance on institutionally imposed peer networks within a comprehensive school setting, mitigating self-selection bias. However, our findings should be interpreted in the context of the study’s limitations. First, while the observed associations were statistically significant, the HRs were relatively small. Therefore, we cannot rule out residual confounding due to unmeasured or inaccurately measured covariates in this cohort study. Second, it is expected that some individuals with underlying mental disorders refrain from seeking help from health care services, which implies that the reported mental disorder diagnoses are likely an underestimate of the true underlying prevalence of these disorders. Third, school class as an indicator for peer network is quite crude and the National Joint Application Register discloses information on school class divisions only for the final year (ie, ninth grade) of comprehensive school. Furthermore, although school classes in Finland are predominantly institutionally imposed without the freedom for pupils to choose their classmates, some schools have classes with special emphasis (eg, music or bilingual teaching) to which pupils are selected based on aptitude tests.^21^ That said, classes within comprehensive schools in the Finnish educational system, particularly during lower secondary education (grades 7-9), are generally stable and transitions between classes are infrequent.^22^ In addition, since Finland is a relatively small and homogeneous Nordic country with a health care system providing universal access to services for all citizens, replications of this study in other countries are necessary to evaluate the generalizability of our findings.

## Conclusions

Based on a nationwide cohort of over 700 000 Finnish individuals, the results of this cohort study suggest a dose-response association between the number of persons with a mental disorder diagnosis in the same peer network during adolescence and later risk of being diagnosed with a mental disorder, even after accounting for a broad set of potential individual and environmental confounders. The association was most clearly present for mood, anxiety, and eating disorders. These findings suggest that mental disorders may be transmitted within adolescent peer networks. Consequently, prevention and intervention measures that consider potential peer influences on early-life mental health could substantially reduce the disease burden of mental disorders in society. Further research is required to clarify the mechanisms that explain these observed associations.

## Notes

Supplement 1. **eTable 1.** Annual Number of Mental Disorder Diagnoses Together With the Number of Exposed/Unexposed Classes and Cohort Members **eTable 2.** Additional Descriptive Statistics of the Study Population **eFigure 1.** Schoenfeld Residuals for the Diagnosis Categories **eTable 3.** Diagnosis-Specific Associations Between Having Ninth Grade Classmates With a Mental Disorder Diagnosis and Later Risk of Being Diagnosed With a Mental Disorder **eFigure 2.** Diagnosis-Specific Associations Between Having Ninth Grade Classmates With a Mental Disorder Diagnosis and Later Risk of Being Diagnosed With a Mental Disorder **eFigure 3.** Diagnosis-Specific Associations Between Having Ninth Grade Classmates With a Mental Disorder Diagnosis and Later Risk of Being Diagnosed With a Mental Disorder Using Binary Exposure **eTable 4.** Sensitivity Analyses on the Associations Between Having Diagnosed Ninth Grade Classmates and Later Risk of Being Diagnosed With a Mental Disorder **eTable 5.** Series of Cox Regression Models Indicating the Respective Confounding Influences of Each Covariate Domain (School-Level, Parental-Level, and Area-Level) **eTable 6.** The Associations Between Having Ninth Grade Classmates With a Mental Disorder Diagnosis and Later Risk of Being Diagnosed With a Mental Disorder Stratified Into Three Shorter Time Periods

Supplement 2.
Data Sharing Statement
