# Supplementary material for: Transmission of Mental Disorders in Adolescent Peer Networks
Source: JAMA Psychiatry. 2024 May 22;81(9):882–8. doi: 10.1001/jamapsychiatry.2024.1126 (PMC11112494; doi:10.1001/jamapsychiatry.2024.1126)
Supplement: Supplement 1. — eTable 1. Annual Number of Mental Disorder Diagnoses Together With the Number of Exposed/Unexposed Classes and Cohort Members eTable 2. Additional Descriptive Statistics of the Study Population eFigure 1. Schoenfeld Residuals for the Diagnosis Categories eTable 3. Diagnosis-Specific Associations Between Having Ninth Grade Classmates With a Mental Disorder Diagnosis and Later Risk of Being Diagnosed With a Mental Disorder eFigure 2. Diagnosis-Specific Associations Between Having Ninth Grade Classmates With a Mental Disorder Diagnosis and Later Risk of Being Diagnosed With a Mental Disorder eFigure 3. Diagnosis-Specific Associations Between Having Ninth Grade Classmates With a Mental Disorder Diagnosis and Later Risk of Being Diagnosed With a Mental Disorder Using Binary Exposure eTable 4. Sensitivity Analyses on the Associations Between Having Diagnosed Ninth Grade Classmates and Later Risk of Being Diagnosed With a Mental Disorder eTable 5. Series of Cox Regression Models Indicating the Respective Confounding Influences of Each Covariate Domain (School-Level, Parental-Level, and Area-Level) eTable 6. The Associations Between Having Ninth Grade Classmates With a Mental Disorder Diagnosis and Later Risk of Being Diagnosed With a Mental Disorder Stratified Into Three Shorter Time Periods [file jamapsychiatry-e241126-s001.pdf]

Supplementary Online Content

Alho J, Gutvilig M, Niemi R, et al. Transmission of mental disorders in adolescent peer networks. *JAMA Psychiatry*. Published online May 22, 2024. doi:10.1001/jamapsychiatry.2024.1126

eTable 1. Annual Number of Mental Disorder Diagnoses Together With the Number of Exposed/Unexposed Classes and Cohort Members

eTable 2. Additional Descriptive Statistics of the Study Population

eFigure 1. Schoenfeld Residuals for the Diagnosis Categories

eTable 3. Diagnosis-Specific Associations Between Having Ninth Grade Classmates With a Mental Disorder Diagnosis and Later Risk of Being Diagnosed With a Mental Disorder

eFigure 2. Diagnosis-Specific Associations Between Having Ninth Grade Classmates With a Mental Disorder Diagnosis and Later Risk of Being Diagnosed With a Mental Disorder

eFigure 3. Diagnosis-Specific Associations Between Having Ninth Grade Classmates With a Mental Disorder Diagnosis and Later Risk of Being Diagnosed With a Mental Disorder Using Binary Exposure

eTable 4. Sensitivity Analyses on the Associations Between Having Diagnosed Ninth Grade Classmates and Later Risk of Being Diagnosed With a Mental Disorder

eTable 5. Series of Cox Regression Models Indicating the Respective Confounding Influences of Each Covariate Domain (School-Level, Parental-Level, and Area-Level)

eTable 6. The Associations Between Having Ninth Grade Classmates With a Mental Disorder Diagnosis and Later Risk of Being Diagnosed With a Mental Disorder Stratified Into Three Shorter Time Periods

This supplementary material has been provided by the authors to give readers additional information about their work.

© 2024 .Alho J et al. *JAMA Psychiatry*.

eTable 1: Annual number of mental disorder diagnoses together with the number of exposed/unexposed classes and cohort members.

Year	Diagnoses before FU No.	Unexposed classes No. (%)	Exposed classes No. (%)	Unexposed individuals No. (%)	Exposed individuals No. (%)
2001	1662	1817 (59.8)	1221 (40.2)	31 673 (60.7)	20 545 (39.3)
2002	2009	1571 (52.4)	1425 (47.6)	27 749 (53.4)	24 233 (46.6)
2003	2233	1528 (50.5)	1495 (49.5)	26 603 (51.9)	24 666 (48.1)
2004	2635	1470 (46.3)	1707 (53.7)	25 575 (47.7)	28 039 (52.3)
2005	2856	1345 (43.2)	1770 (56.8)	23 444 (44.3)	29 488 (55.7)
2006	3244	1247 (38.9)	1956 (61.1)	21 932 (40.4)	32 294 (59.6)
2007	3531	1160 (36.6)	2010 (63.4)	20 393 (38.2)	32 965 (61.8)
2008	3854	1045 (33.0)	2121 (67.0)	17 940 (34.2)	34 572 (65.8)
2009	4317	868 (27.9)	2244 (72.1)	15 198 (29.3)	36 674 (70.7)
2010	4583	783 (25.3)	2312 (74.7)	13 753 (26.8)	37 531 (73.2)
2011	5012	682 (22.2)	2386 (77.8)	11 742 (23.4)	38 362 (76.6)
2012	5368	516 (17.7)	2403 (82.3)	8734 (18.8)	37 787 (81.2)
2013	6129	438 (15.0)	2473 (85.0)	7207 (16.2)	37 277 (83.8)

Number of any of the examined mental disorder diagnoses (ICD-10 F10–F50 or F90–F98) before follow-up (FU) shown for each year of the study period (i.e., years when the cohort members were on the ninth grade) together with the number of school classes without and with diagnosed cohort members (unexposed and exposed classes) and number of cohort members without and with diagnosed classmates (unexposed and exposed individuals).

© 2024 .Alho J et al. *JAMA Psychiatry*.

eTable 2: Additional descriptive statistics of the study population.

	No. (%) or mean (SD)
Diagnoses in class (exposure)	
None	251 943 (37.8)
One	215 384 (32.3)
More than one	199 049 (29.9)
School class size	19.8 (3.6)
School's ninth grade size	111.2 (45.3)
Regional urbanicity	
Unknown	5454 (0.8)
Urban	377 422 (52.9)
Semi-urban	133 226 (18.7)
Rural	197 707 (27.6)
Regional morbidity	
Quintile 1 (lowest)	143 711 (20.1)
Quintile 2	141 987 (19.9)
Quintile 3	142 625 (20.0)
Quintile 4	143 190 (20.0)
Quintile 5 (highest)	142 296 (19.9)
Regional employment	
Quintile 1 (highest)	148 055 (20.7)
Quintile 2	137 479 (19.3)
Quintile 3	140 203 (19.6)
Quintile 4	140 028 (19.6)
Quintile 5 (lowest)	148 044 (20.7)
Regional education	
Quintile 1 (highest)	131 509 (18.4)
Quintile 2	141 160 (19.8)
Quintile 3	154 947 (21.7)
Quintile 4	146 872 (20.6)
Quintile 5 (lowest)	139 321 (19.5)
Mother's education level	
Primary	97 907 (13.7)
Secondary	306 798 (43.0)
High	309 104 (43.3)
Father's education level	
Primary	165 589 (23.2)
Secondary	309 045 (43.3)
High	239 175 (33.5)
Mother's income level	
Unknown	9177 (1.3)
Quintile 1 (lowest)	140 927 (19.7)
Quintile 2	140 926 (19.7)
Quintile 3	140 927 (19.7)
Quintile 4	140 926 (19.7)
Quintile 5 (highest)	140 926 (19.7)
Father's income level	
Unknown	34 168 (4.8)
Quintile 1 (lowest)	135 929 (19.0)
Quintile 2	135 928 (19.0)
Quintile 3	135 932 (19.0)
Quintile 4	135 924 (19.0)
Quintile 5 (highest)	135 928 (19.0)
Mother's mental disorder	
No	645 548 (90.4)
Yes	68 261 (9.6)
Father's mental disorder	
No	640 030 (89.7)
Yes	73 779 (10.3)

© 2024 .Alho J et al. *JAMA Psychiatry*.

eFigure 1: Schoenfeld residuals for the diagnosis categories.

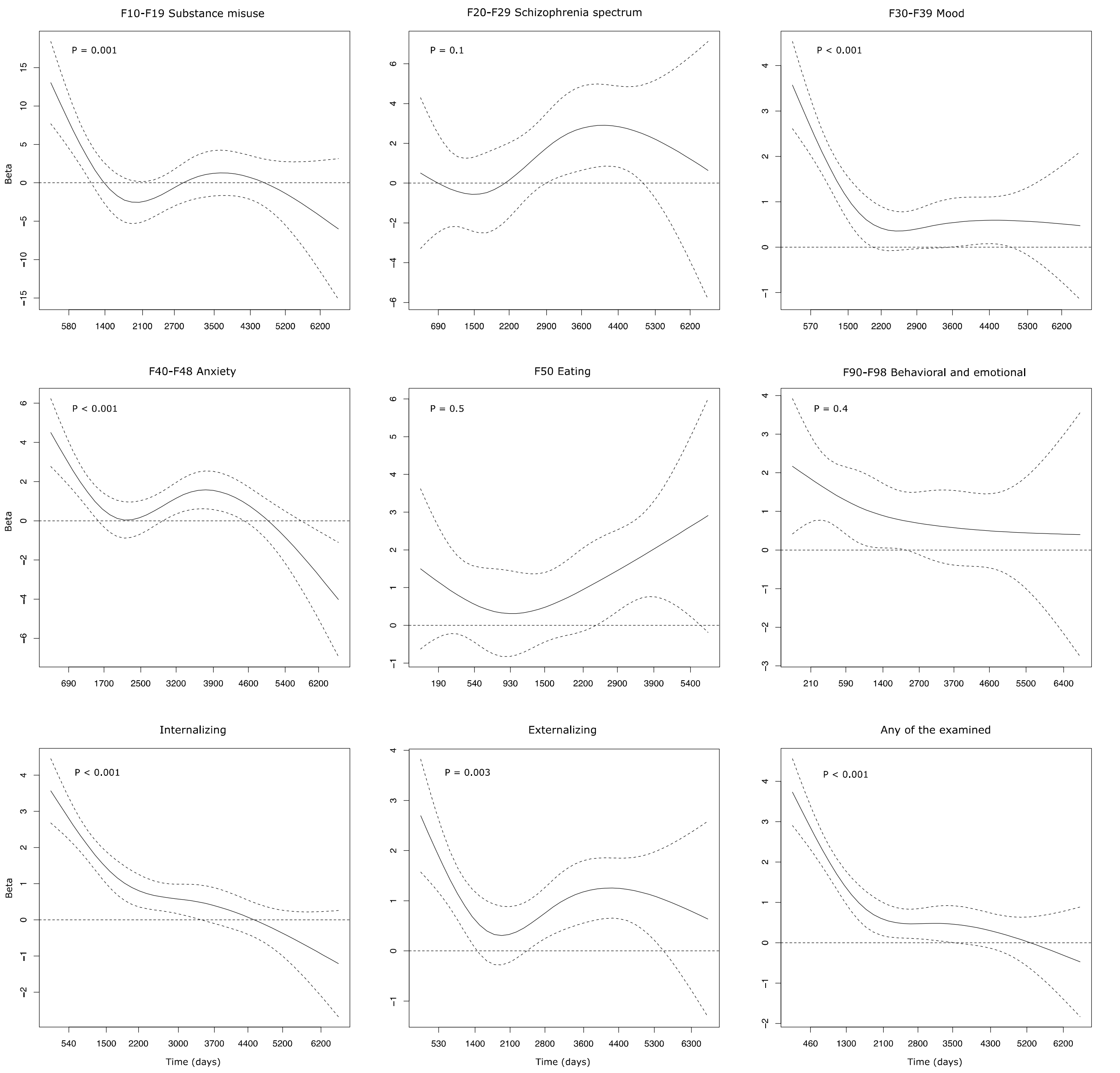

The graph depicts the combined coefficients of the exposure variable (i.e., one or more diagnosed classmates) as a function of follow-up time. P value from Schoenfeld test is shown in each plot (P < 0.05 indicates a failure to meet the proportional hazards assumption). The Cox models were adjusted for sex, birth year, school class size, school's ninth grade size, area-level urbanicity, area-level morbidity, area-level education level, area-level employment rate, parental education, parental income, and parental mental health, with a random intercept per school.

© 2024 .Alho J et al. *JAMA Psychiatry*.

eTable 3: Diagnosis-specific associations between having ninth grade classmates with a mental disorder diagnosis and later risk of being diagnosed with a mental disorder.

				Diagnoses in class (exposure)						
				None (ref)	One			More than one		
	**Mental disorder**	**No.**	**IR**	**No.**	**No.**	**HR (95% CI)**	**P**	**No.**	**HR (95% CI)**	**P**
First year	Substance misuse disorders	1679	252	1488	174	1.21 (1.03 to 1.42)	**0.020**	17	1.02 (0.63 to 1.65)	0.942
	Schizophr. spectr. disorders	339	51	NA	NA	1.13 (0.60 to 2.12)	0.713	NA	NA	NA
	Mood disorders	4382	660	3029	1019	1.16 (1.08 to 1.25)	**<0.001**	334	1.40 (1.25 to 1.57)	**<0.001**
	Anxiety disorders	4890	737	3184	1262	1.10 (1.03 to 1.17)	**0.006**	444	1.21 (1.09 to 1.34)	**<0.001**
	Eating disorders	1109	167	965	129	1.18 (0.98 to 1.42)	0.077	15	1.55 (0.93 to 2.58)	0.096
	Behav./emotional disorders	2093	315	1026	642	1.04 (0.94 to 1.15)	0.485	425	1.14 (1.01 to 1.29)	**0.038**
	Internalizing disorders	8498	1284	4109	2691	1.10 (1.05 to 1.16)	**<0.001**	1698	1.21 (1.14 to 1.28)	**<0.001**
	Externalizing disorders	2191	329	1451	526	1.09 (0.99 to 1.21)	0.091	214	1.38 (1.19 to 1.60)	**<0.001**
	Any of the above	11 542	1747	3331	3665	1.09 (1.04 to 1.14)	**<0.001**	4546	1.18 (1.13 to 1.24)	**<0.001**
Years 2 and 3	Substance misuse disorders	3993	204	3592	363	1.18 (1.06 to 1.32)	**0.003**	38	1.30 (0.94 to 1.80)	0.110
	Schizophr. spectr. disorders	1142	58	NA	NA	0.85 (0.57 to 1.27)	0.432	NA	NA	NA
	Mood disorders	10 385	532	7454	2313	1.08 (1.03 to 1.13)	**0.002**	618	1.08 (0.99 to 1.17)	0.079
	Anxiety disorders	12 288	630	8219	3103	1.04 (1.00 to 1.09)	0.054	966	1.05 (0.98 to 1.12)	0.195
	Eating disorders	1791	91	1611	165	0.97 (0.83 to 1.14)	0.722	15	1.02 (0.61 to 1.70)	0.944
	Behav./emotional disorders	2698	138	1317	854	1.07 (0.98 to 1.17)	0.136	527	1.11 (1.00 to 1.24)	0.052
	Internalizing disorders	19 432	1000	9759	6130	1.04 (1.01 to 1.08)	**0.010**	3543	1.05 (1.01 to 1.10)	**0.013**
	Externalizing disorders	4504	230	3126	1026	1.01 (0.94 to 1.09)	0.745	352	1.14 (1.02 to 1.28)	**0.024**
	Any of the above	24 469	1262	7497	7658	1.00 (0.97 to 1.03)	0.936	9314	1.08 (1.04 to 1.11)	**<0.001**
Years 4 and 5	Substance misuse disorders	4455	142	4094	329	1.02 (0.91 to 1.14)	0.705	32	1.10 (0.78 to 1.56)	0.584
	Schizophr. spectr. disorders	1233	39	NA	NA	0.97 (0.67 to 1.39)	0.853	NA	NA	NA
	Mood disorders	11 278	360	8181	2406	1.06 (1.01 to 1.11)	**0.021**	691	1.18 (1.09 to 1.27)	**<0.001**
	Anxiety disorders	14 799	473	10 080	3600	1.04 (1.00 to 1.08)	**0.035**	1119	1.07 (1.01 to 1.14)	**0.033**
	Eating disorders	988	31	872	107	1.17 (0.96 to 1.44)	0.126	9	1.13 (0.58 to 2.20)	0.713
	Behav./emotional disorders	881	28	430	284	1.10 (0.95 to 1.29)	0.209	167	1.17 (0.97 to 1.41)	0.104
	Internalizing disorders	21 998	704	11 169	6778	1.05 (1.02 to 1.08)	**0.004**	4051	1.13 (1.09 to 1.17)	**<0.001**
	Externalizing disorders	4729	151	3357	1049	1.04 (0.97 to 1.11)	0.301	323	1.13 (1.00 to 1.27)	**0.044**
	Any of the above	26 260	842	8176	8443	1.05 (1.02 to 1.08)	**0.003**	9641	1.10 (1.07 to 1.14)	**<0.001**
After year 5	Substance misuse disorders	15 776	210	14 676	1013	0.97 (0.91 to 1.04)	0.400	87	1.06 (0.86 to 1.31)	0.581
	Schizophr. spectr. disorders	4671	62	NA	NA	1.26 (1.06 to 1.50)	**0.008**	NA	NA	NA
	Mood disorders	46 831	633	36 452	8536	1.03 (1.00 to 1.05)	**0.018**	1843	1.06 (1.01 to 1.12)	**0.010**
	Anxiety disorders	73 828	1010	56 775	14 039	1.02 (1.00 to 1.04)	**0.027**	3014	1.01 (0.97 to 1.05)	0.662
	Eating disorders	2340	31	2084	233	1.19 (1.04 to 1.37)	**0.012**	23	1.44 (0.95 to 2.17)	0.085
	Behav./emotional disorders	4539	60	2849	1214	1.02 (0.95 to 1.09)	0.534	476	1.05 (0.95 to 1.17)	0.308
	Internalizing disorders	94 819	1316	57 087	26 503	1.02 (1.01 to 1.04)	**0.003**	11 229	1.03 (1.01 to 1.06)	**0.003**
	Externalizing disorders	17 714	236	13 511	3361	1.04 (1.00 to 1.08)	0.057	842	1.16 (1.08 to 1.25)	**<0.001**
	Any of the above	104 956	1467	43 310	34 094	1.00 (0.99 to 1.02)	0.522	27 552	1.03 (1.01 to 1.05)	**<0.001**

Incidence rates (IRs), hazard ratios (HRs) with 95% confidence intervals, and P-values shown for the associations in four follow-up time windows. IR was calculated per 100 000 person-years at risk. For the calculation of the HRs, the Cox models were adjusted for sex, birth year, school class size, school's ninth grade size, area-level urbanicity, area-level morbidity, area-level education level, area-level employment rate, parental education, parental income, and parental mental health, with a random intercept per school. Results of analyses with less than or equal to three participants are not reported due to data protection regulations. See also eFigure 2.

© 2024 .Alho J et al. *JAMA Psychiatry*.

eFigure 2: Diagnosis-specific associations between having ninth grade classmates with a mental disorder diagnosis and later risk of being diagnosed with a mental disorder.

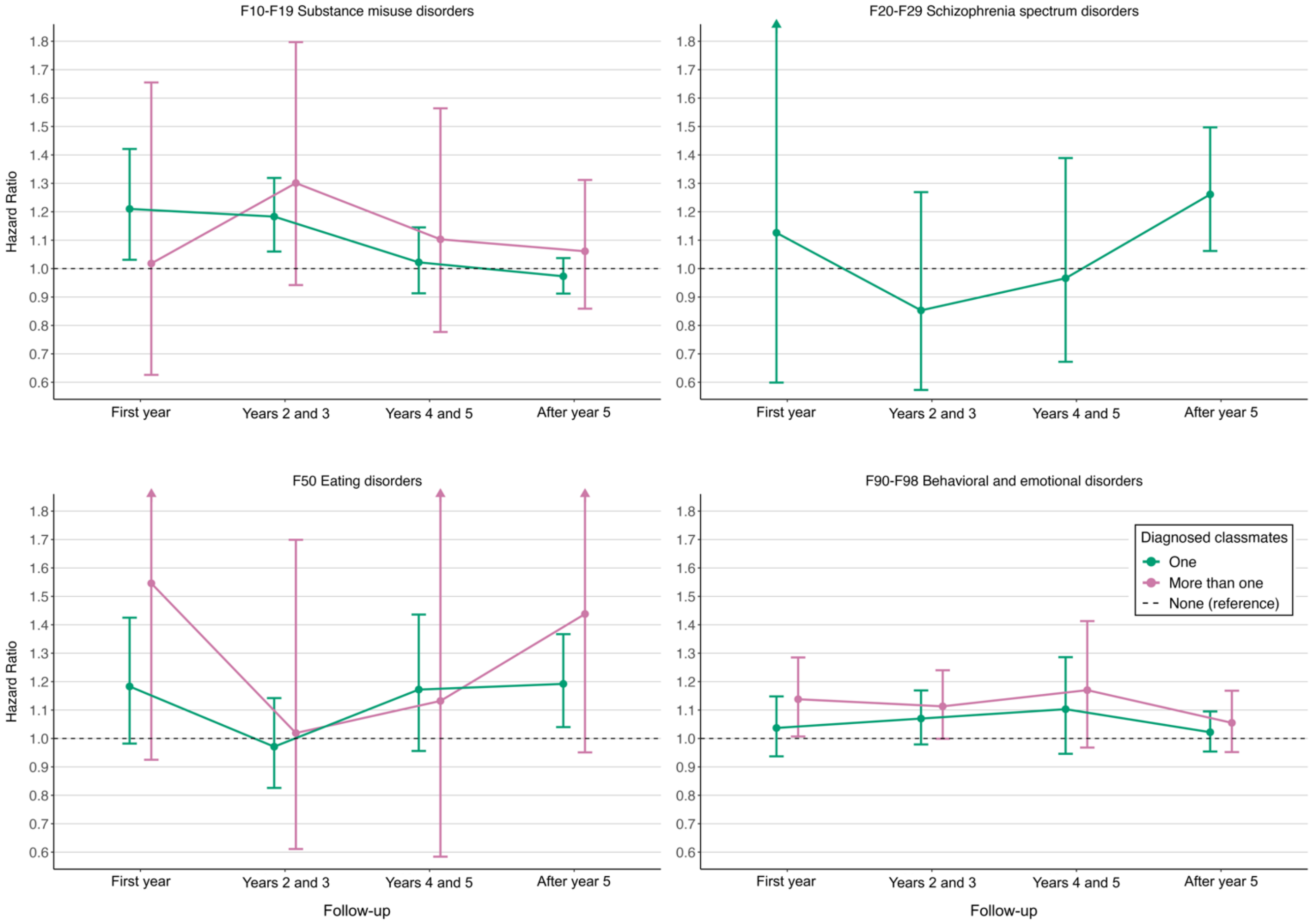

Hazard ratios with 95% confidence intervals shown for substance misuse, schizophrenia spectrum, eating, and behavioral and emotional disorders in four follow-up time windows. The arrow indicates that the confidence interval extends outside the range of the graph. The Cox models were adjusted for sex, birth year, school class size, school's ninth grade size, area-level urbanicity, area-level morbidity, area-level education level, area-level employment rate, parental education, parental income, and parental mental health, with a random intercept per school. Note that the number of individuals who received a schizophrenia spectrum diagnosis during follow-up and who had more than one classmate with a schizophrenia spectrum diagnosis was too low to be reported due to data protection regulations (see eTable 2). For the diagnosis-specific associations for mood, anxiety, internalizing, and externalizing disorders, see Figure 2 of the main text.

© 2024 .Alho J et al. *JAMA Psychiatry*.

eFigure 3: Diagnosis-specific associations between having ninth grade classmates with a mental disorder diagnosis and later risk of being diagnosed with a mental disorder using binary exposure.

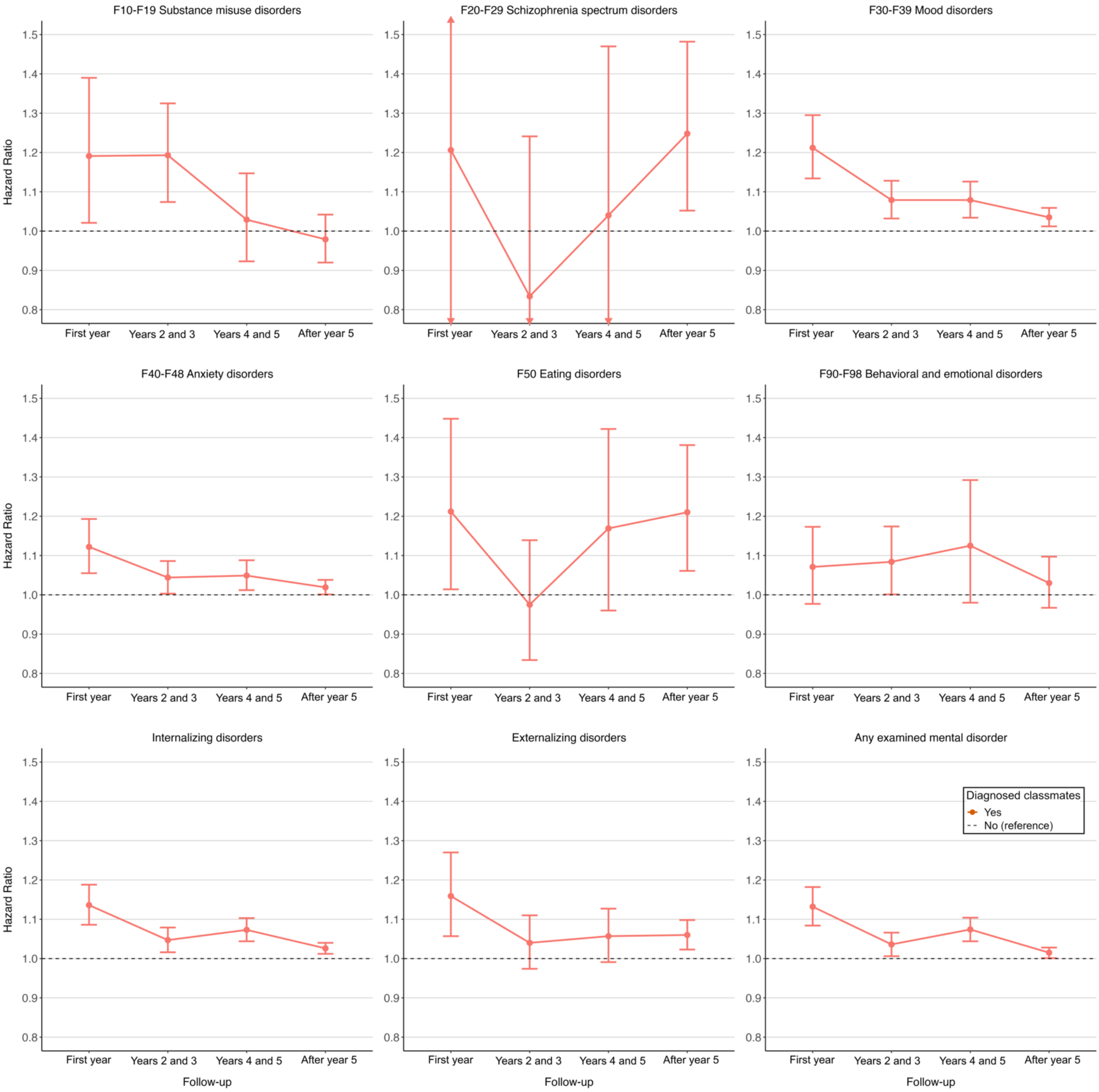

Hazard ratios with 95% confidence intervals shown for all the examined diagnostic categories in four follow-up time windows. The arrow indicates that the confidence interval extends outside the range of the graph. The Cox models were adjusted for sex, birth year, school class size, school's ninth grade size, area-level urbanicity, area-level morbidity, area-level education level, area-level employment rate, parental education, parental income, and parental mental health, with a random intercept per school.

© 2024 .Alho J et al. *JAMA Psychiatry*.

eTable 4: Sensitivity analyses on the associations between having diagnosed ninth grade classmates and later risk of being diagnosed with a mental disorder.

a

		Diagnoses in class (exposure)			
		None (ref)	Yes		
	**Mental disorder**	**No.**	**No.**	**HR (95% CI)**	**P**
Entire follow-up	Substance misuse disorders	28 510	2429	1.02 (0.98 to 1.07)	0.306
	Schizophr. spectr. disorders	9646	278	1.11 (0.99 to 1.25)	0.081
	Mood disorders	61 845	20 216	1.05 (1.03 to 1.07)	**<0.001**
	Anxiety disorders	86 401	30 676	1.03 (1.01 to 1.04)	**<0.001**
	Eating disorders	6515	818	1.12 (1.04 to 1.20)	**0.004**
	Behav./emotional disorders	6963	5727	1.05 (1.01 to 1.09)	**0.012**
	Internalizing disorders	84 562	64 681	1.04 (1.03 to 1.05)	**<0.001**
	Externalizing disorders	25 552	9299	1.05 (1.02 to 1.08)	**<0.001**
	Any of the above	62 314	104 913	1.03 (1.02 to 1.04)	**<0.001**
First year	Substance misuse disorders	1488	191	1.19 (1.02 to 1.39)	**0.026**
	Schizophr. spectr. disorders	328	11	1.21 (0.66 to 2.20)	0.542
	Mood disorders	3029	1353	1.21 (1.13 to 1.29)	**<0.001**
	Anxiety disorders	3184	1706	1.12 (1.05 to 1.19)	**<0.001**
	Eating disorders	965	144	1.21 (1.01 to 1.45)	**0.034**
	Behav./emotional disorders	1026	1067	1.07 (0.98 to 1.17)	0.142
	Internalizing disorders	4109	4389	1.14 (1.09 to 1.19)	**<0.001**
	Externalizing disorders	1451	740	1.16 (1.06 to 1.27)	**0.002**
	Any of the above	3331	8211	1.13 (1.08 to 1.18)	**<0.001**
Years 2 and 3	Substance misuse disorders	3592	401	1.19 (1.07 to 1.32)	**<0.001**
	Schizophr. spectr. disorders	1117	25	0.83 (0.56 to 1.24)	0.371
	Mood disorders	7454	2931	1.08 (1.03 to 1.13)	**<0.001**
	Anxiety disorders	8219	4069	1.04 (1.00 to 1.09)	**0.034**
	Eating disorders	1611	180	0.97 (0.83 to 1.14)	0.748
	Behav./emotional disorders	1317	1381	1.08 (1.00 to 1.17)	**0.048**
	Internalizing disorders	9759	9673	1.05 (1.02 to 1.08)	**0.002**
	Externalizing disorders	3126	1378	1.04 (0.97 to 1.11)	0.237
	Any of the above	7497	16 972	1.04 (1.01 to 1.07)	**0.018**
Years 4 and 5	Substance misuse disorders	4094	361	1.03 (0.92 to 1.15)	0.610
	Schizophr. spectr. disorders	1200	33	1.04 (0.73 to 1.47)	0.827
	Mood disorders	8181	3097	1.08 (1.03 to 1.13)	**<0.001**
	Anxiety disorders	10 080	4719	1.05 (1.01 to 1.09)	**0.009**
	Eating disorders	872	116	1.17 (0.96 to 1.42)	0.120
	Behav./emotional disorders	430	451	1.12 (0.98 to 1.29)	0.096
	Internalizing disorders	11 169	10 829	1.07 (1.04 to 1.10)	**<0.001**
	Externalizing disorders	3357	1372	1.06 (0.99 to 1.13)	0.093
	Any of the above	8176	18 084	1.07 (1.04 to 1.10)	**<0.001**
After year 5	Substance misuse disorders	14676	1100	0.98 (0.92 to 1.04)	0.520
	Schizophr. spectr. disorders	4535	136	1.25 (1.05 to 1.48)	**0.011**
	Mood disorders	36 452	10 379	1.04 (1.01 to 1.06)	**0.003**
	Anxiety disorders	56 775	17 053	1.02 (1.00 to 1.04)	**0.034**
	Eating disorders	2084	256	1.21 (1.06 to 1.38)	**0.004**
	Behav./emotional disorders	2849	1690	1.03 (0.97 to 1.10)	0.357
	Internalizing disorders	57 087	37 732	1.03 (1.01 to 1.04)	**<0.001**
	Externalizing disorders	13 511	4203	1.06 (1.02 to 1.10)	**0.001**
	Any of the above	43 310	61 646	1.01 (1.00 to 1.03)	**0.030**

a: Hazard ratios (HRs) with 95% confidence intervals (CIs) and P-values shown for the associations using binary exposure. Results are shown for the entire follow-up and separately in four shorter time windows. The Cox models were adjusted for sex, birth year, school class size, school's ninth grade size, area-level urbanicity, area-level morbidity, area-level education level, area-level employment rate, parental education, parental income, and parental mental health, with a random intercept per school.

© 2024 .Alho J et al. *JAMA Psychiatry*.

b

		Diagnoses in class (exposure)			
		None (ref)	Yes		
	**Mental disorder**	**No.**	**No.**	**HR (95% CI)**	**P**
Entire follow-up	Substance misuse disorders	28 903	2036	1.00 (0.95 to 1.04)	0.936
	Schizophr. spectr. disorders	9713	211	1.17 (1.02 to 1.34)	**0.023**
	Mood disorders	66 459	15 602	1.04 (1.02 to 1.05)	**<0.001**
	Anxiety disorders	95 413	21 664	1.02 (1.01 to 1.04)	**0.004**
	Eating disorders	6694	639	1.12 (1.03 to 1.21)	**0.008**
	Behav./emotional disorders	10 439	2251	1.07 (1.02 to 1.12)	**0.006**
	Internalizing disorders	102 263	46 980	1.03 (1.01 to 1.04)	**<0.001**
	Externalizing disorders	30 113	4738	1.03 (1.00 to 1.06)	0.068
	Any of the above	97 243	69 984	1.02 (1.01 to 1.03)	**0.002**
First year	Substance misuse disorders	1515	164	1.20 (1.02 to 1.42)	**0.029**
	Schizophr. spectr. disorders	333	6	0.92 (0.41 to 2.08)	0.849
	Mood disorders	3321	1061	1.21 (1.13 to 1.30)	**<0.001**
	Anxiety disorders	3660	1230	1.13 (1.05 to 1.21)	**<0.001**
	Eating disorders	997	112	1.21 (1.00 to 1.48)	0.054
	Behav./emotional disorders	1626	467	1.18 (1.06 to 1.32)	**0.002**
	Internalizing disorders	5273	3225	1.12 (1.07 to 1.17)	**<0.001**
	Externalizing disorders	1825	366	1.14 (1.02 to 1.28)	**0.025**
	Any of the above	5954	5588	1.09 (1.05 to 1.13)	**<0.001**
Years 2 and 3	Substance misuse disorders	3663	330	1.15 (1.03 to 1.29)	**0.014**
	Schizophr. spectr. disorders	1121	21	1.00 (0.65 to 1.54)	0.998
	Mood disorders	8135	2250	1.07 (1.02 to 1.12)	**0.009**
	Anxiety disorders	9382	2906	1.06 (1.02 to 1.11)	**0.007**
	Eating disorders	1646	145	1.02 (0.86 to 1.21)	0.796
	Behav./emotional disorders	2148	550	1.09 (0.99 to 1.20)	0.070
	Internalizing disorders	12 436	6996	1.03 (1.00 to 1.07)	**0.037**
	Externalizing disorders	3852	652	1.03 (0.95 to 1.12)	0.510
	Any of the above	13 072	11 397	1.04 (1.01 to 1.07)	**0.003**
Years 4 and 5	Substance misuse disorders	4145	310	1.04 (0.92 to 1.16)	0.547
	Schizophr. spectr. disorders	1209	24	1.07 (0.72 to 1.61)	0.735
	Mood disorders	8910	2368	1.07 (1.02 to 1.12)	**0.006**
	Anxiety disorders	11 517	3282	1.03 (0.99 to 1.08)	0.115
	Eating disorders	900	88	1.14 (0.91 to 1.42)	0.262
	Behav./emotional disorders	703	178	1.16 (0.98 to 1.38)	0.078
	Internalizing disorders	14 197	7801	1.06 (1.03 to 1.09)	**<0.001**
	Externalizing disorders	4053	676	1.07 (0.98 to 1.16)	0.131
	Any of the above	14357	11 903	1.04 (1.01 to 1.06)	**0.007**
After year 5	Substance misuse disorders	14 854	922	0.95 (0.89 to 1.02)	0.135
	Schizophr. spectr. disorders	4566	105	1.30 (1.07 to 1.58)	**0.009**
	Mood disorders	38 778	8053	1.02 (0.99 to 1.04)	0.131
	Anxiety disorders	61 770	12 058	1.01 (0.99 to 1.04)	0.167
	Eating disorders	2139	201	1.19 (1.03 to 1.38)	**0.019**
	Behav./emotional disorders	3922	617	0.98 (0.90 to 1.07)	0.632
	Internalizing disorders	67 342	27 477	1.02 (1.00 to 1.03)	**0.038**
	Externalizing disorders	15 452	2262	1.03 (0.98 to 1.07)	0.268
	Any of the above	63 860	41 096	1.01 (0.99 to 1.02)	0.354

b: Hazard ratios (HRs) with 95% confidence intervals (CIs) and P-values shown for the associations using binary exposure and limiting exposure diagnoses to three years preceding start of follow-up. Results are shown for the entire follow-up and separately in four shorter time windows. The Cox models were adjusted for sex, birth year, school class size, school's ninth grade size, area-level urbanicity, area-level morbidity, area-level education level, area-level employment rate, parental education, parental income, and parental mental health, with a random intercept per school.

© 2024 .Alho J et al. *JAMA Psychiatry*.

c

Period	Mental disorder	None (ref) No.	Yes No.	Yes HR (95% CI)	Yes P
Entire follow-up	Substance misuse disorders	26 780	2251	1.02 (0.98 to 1.07)	0.332
	Schizophr. spectr. disorders	8991	252	1.14 (1.00 to 1.29)	**0.046**
	Mood disorders	58 402	18 538	1.04 (1.03 to 1.06)	**<0.001**
	Anxiety disorders	81 546	28 361	1.02 (1.01 to 1.04)	**0.002**
	Eating disorders	6119	731	1.12 (1.03 to 1.21)	**0.005**
	Behav./emotional disorders	6585	5227	1.03 (0.99 to 1.07)	0.135
	Internalizing disorders	80 258	59 851	1.03 (1.02 to 1.04)	**<0.001**
	Externalizing disorders	24 166	8480	1.04 (1.01 to 1.06)	**0.005**
	Any of the above	59 510	97 456	1.02 (1.01 to 1.03)	**<0.001**
First year	Substance misuse disorders	1407	183	1.22 (1.04 to 1.42)	**0.015**
	Schizophr. spectr. disorders	307	10	1.22 (0.65 to 2.30)	0.536
	Mood disorders	2877	1252	1.21 (1.13 to 1.29)	**<0.001**
	Anxiety disorders	3023	1585	1.11 (1.04 to 1.18)	**0.002**
	Eating disorders	912	135	1.26 (1.05 to 1.51)	**0.013**
	Behav./emotional disorders	980	975	1.03 (0.94 to 1.14)	0.499
	Internalizing disorders	3923	4066	1.12 (1.07 to 1.17)	**<0.001**
	Externalizing disorders	1373	679	1.14 (1.04 to 1.25)	**0.007**
	Any of the above	3186	7662	1.13 (1.08 to 1.18)	**<0.001**
Years 2 and 3	Substance misuse disorders	3395	369	1.18 (1.06 to 1.32)	**0.003**
	Schizophr. spectr. disorders	1030	23	0.87 (0.57 to 1.31)	0.502
	Mood disorders	7048	2719	1.09 (1.04 to 1.14)	**<0.001**
	Anxiety disorders	7810	3804	1.04 (1.00 to 1.08)	0.068
	Eating disorders	1519	159	0.96 (0.81 to 1.13)	0.606
	Behav./emotional disorders	1237	1267	1.08 (1.00 to 1.18)	0.063
	Internalizing disorders	9304	9006	1.04 (1.01 to 1.08)	**0.007**
	Externalizing disorders	2972	1258	1.03 (0.96 to 1.10)	0.455
	Any of the above	7172	15 860	1.03 (1.00 to 1.06)	**0.029**
Years 4 and 5	Substance misuse disorders	3856	334	1.02 (0.91 to 1.15)	0.680
	Schizophr. spectr. disorders	1121	32	1.13 (0.80 to 1.61)	0.494
	Mood disorders	7765	2870	1.08 (1.03 to 1.12)	**0.001**
	Anxiety disorders	9578	4394	1.04 (1.00 to 1.08)	**0.046**
	Eating disorders	820	107	1.21 (0.99 to 1.48)	0.069
	Behav./emotional disorders	408	410	1.09 (0.94 to 1.25)	0.253
	Internalizing disorders	10 687	10 062	1.06 (1.03 to 1.09)	**<0.001**
	Externalizing disorders	3183	1265	1.05 (0.98 to 1.13)	0.133
	Any of the above	7855	16 889	1.06 (1.03 to 1.09)	**<0.001**
After year 5	Substance misuse disorders	13 756	1020	0.98 (0.92 to 1.04)	0.530
	Schizophr. spectr. disorders	4231	124	1.29 (1.07 to 1.54)	**0.006**
	Mood disorders	34 343	9445	1.03 (1.00 to 1.05)	**0.032**
	Anxiety disorders	53 439	15 692	1.02 (1.00 to 1.04)	0.064
	Eating disorders	1944	226	1.22 (1.06 to 1.40)	**0.006**
	Behav./emotional disorders	2674	1529	1.02 (0.95 to 1.09)	0.633
	Internalizing disorders	54 023	34 796	1.02 (1.01 to 1.04)	**0.002**
	Externalizing disorders	12 751	3812	1.04 (1.01 to 1.08)	**0.020**
	Any of the above	41 297	57 045	1.01 (1.00 to 1.03)	0.097

Column header: Diagnoses in class (exposure) — None (ref) / Yes.

c: Hazard ratios (HRs) with 95% confidence intervals (CIs) and P-values shown for the associations using binary exposure and limiting school class sizes within 5th and 95th percentiles (i.e., classes of 12 to 25 pupils). Results are shown for the entire follow-up and separately in four shorter time windows. The Cox models were adjusted for sex, birth year, school class size, school's ninth grade size, area-level urbanicity, area-level morbidity, area-level education level, area-level employment rate, parental education, parental income, and parental mental health, with a random intercept per school.

© 2024 .Alho J et al. *JAMA Psychiatry*.

eTable 5: Series of Cox regression models indicating the respective confounding influences of each covariate domain (school-level, parental-level, and area-level).

	Mental disorder	Model 1	Model 2	Model 3	Model 4	Model 5	Model 6
First year of follow-up	F10-F19 Substance misuse disorders	1.38 (1.18 to 1.60)	1.23 (1.05 to 1.43)	1.24 (1.06 to 1.44)	1.21 (1.04 to 1.41)	1.20 (1.03 to 1.40)	1.19 (1.02 to 1.39)
	F20-F29 Schizophrenia spectrum disorders	1.27 (0.70 to 2.32)	1.24 (0.68 to 2.27)	1.25 (0.68 to 2.28)	1.23 (0.67 to 2.24)	1.22 (0.67 to 2.22)	1.21 (0.66 to 2.20)
	F30-F39 Mood disorders	1.32 (1.23 to 1.41)	1.24 (1.16 to 1.32)	1.24 (1.16 to 1.32)	1.23 (1.15 to 1.31)	1.22 (1.14 to 1.30)	1.21 (1.13 to 1.29)
	F40-F48 Anxiety disorders	1.20 (1.13 to 1.27)	1.14 (1.07 to 1.21)	1.14 (1.07 to 1.22)	1.14 (1.07 to 1.21)	1.12 (1.05 to 1.19)	1.12 (1.05 to 1.19)
	F50 Eating disorders	1.32 (1.10 to 1.57)	1.31 (1.10 to 1.56)	1.28 (1.08 to 1.53)	1.27 (1.06 to 1.51)	1.23 (1.03 to 1.47)	1.21 (1.01 to 1.45)
	F90-F98 Behavioral and emotional disorders	1.12 (1.02 to 1.23)	1.09 (0.99 to 1.19)	1.09 (1.00 to 1.20)	1.07 (0.98 to 1.18)	1.08 (0.99 to 1.18)	1.07 (0.98 to 1.17)
	Internalizing disorders	1.23 (1.18 to 1.28)	1.15 (1.10 to 1.21)	1.16 (1.11 to 1.21)	1.15 (1.10 to 1.20)	1.14 (1.09 to 1.19)	1.14 (1.09 to 1.19)
	Externalizing disorders	1.27 (1.16 to 1.39)	1.20 (1.09 to 1.31)	1.20 (1.10 to 1.32)	1.17 (1.07 to 1.28)	1.18 (1.08 to 1.30)	1.16 (1.06 to 1.27)
	Any of the above	1.22 (1.17 to 1.27)	1.15 (1.10 to 1.20)	1.15 (1.10 to 1.20)	1.14 (1.09 to 1.19)	1.13 (1.09 to 1.18)	1.13 (1.08 to 1.18)
Entire follow-up	F10-F19 Substance misuse disorders	1.08 (1.04 to 1.13)	1.03 (0.99 to 1.08)	1.04 (1.00 to 1.08)	1.02 (0.98 to 1.07)	1.03 (0.99 to 1.07)	1.02 (0.98 to 1.07)
	F20-F29 Schizophrenia spectrum disorders	1.15 (1.02 to 1.29)	1.12 (1.00 to 1.27)	1.12 (1.00 to 1.27)	1.11 (0.99 to 1.25)	1.12 (0.99 to 1.26)	1.11 (0.99 to 1.25)
	F30-F39 Mood disorders	1.09 (1.08 to 1.11)	1.06 (1.04 to 1.07)	1.06 (1.04 to 1.08)	1.05 (1.03 to 1.07)	1.05 (1.04 to 1.07)	1.05 (1.03 to 1.07)
	F40-F48 Anxiety disorders	1.05 (1.04 to 1.07)	1.03 (1.01 to 1.04)	1.03 (1.02 to 1.04)	1.03 (1.01 to 1.04)	1.03 (1.01 to 1.04)	1.03 (1.01 to 1.04)
	F50 Eating disorders	1.19 (1.11 to 1.28)	1.16 (1.07 to 1.25)	1.14 (1.06 to 1.23)	1.14 (1.06 to 1.23)	1.13 (1.05 to 1.22)	1.12 (1.04 to 1.20)
	F90-F98 Behavioral and emotional disorders	1.07 (1.03 to 1.11)	1.06 (1.02 to 1.10)	1.06 (1.02 to 1.10)	1.05 (1.01 to 1.09)	1.05 (1.02 to 1.09)	1.05 (1.01 to 1.09)
	Internalizing disorders	1.07 (1.06 to 1.08)	1.04 (1.03 to 1.05)	1.04 (1.03 to 1.05)	1.04 (1.03 to 1.05)	1.04 (1.03 to 1.05)	1.04 (1.03 to 1.05)
	Externalizing disorders	1.11 (1.09 to 1.14)	1.07 (1.04 to 1.10)	1.07 (1.05 to 1.10)	1.05 (1.02 to 1.08)	1.07 (1.04 to 1.09)	1.05 (1.02 to 1.08)
	Any of the above	1.06 (1.05 to 1.07)	1.03 (1.02 to 1.04)	1.03 (1.02 to 1.04)	1.03 (1.02 to 1.04)	1.03 (1.02 to 1.04)	1.03 (1.02 to 1.04)

Hazard ratios (and 95% confidence intervals) shown for the first year of follow-up and entire follow-up separately.

Model 1: adjusted for sex and birth year.

Model 2: adjusted for sex and birth year, with a random intercept per school.

Model 3: adjusted for sex, birth year, school class size, and school’s ninth grade size, with a random intercept per school.

Model 4: adjusted for sex, birth year, parental education, parental income, and parental mental health, with a random intercept per school.

Model 5: adjusted for sex, birth year, area-level urbanicity, area-level morbidity, area-level education level, and area-level employment rate, with a random intercept per school.

Model 6: adjusted for sex, birth year, school class size, school grade size, parental education, parental income, parental mental health, area-level urbanicity, area-level morbidity, area-level education level, and area-level employment rate, with a random intercept per school.

© 2024 .Alho J et al. *JAMA Psychiatry*.

eTable 6: The associations between having ninth grade classmates with a mental disorder diagnosis and later risk of being diagnosed with a mental disorder stratified into three shorter time periods.

	One diagnosed classmate		More than one diagnosed classmate	
Period	Model 1	Model 2	Model 1	Model 2
2001–2004	1.02 (1.00 to 1.04)	1.01 (0.99 to 1.03)	1.08 (1.06 to 1.11)	1.04 (1.02 to 1.07)
2005–2008	1.03 (1.01 to 1.05)	1.01 (0.99 to 1.03)	1.11 (1.08 to 1.13)	1.06 (1.04 to 1.08)
2009–2013	1.04 (1.01 to 1.06)	1.03 (1.00 to 1.05)	1.10 (1.08 to 1.13)	1.07 (1.05 to 1.09)

Hazard ratios (and 95% confidence intervals) shown for the associations between having one or more than one ninth grade classmate with any of the examined mental disorder diagnoses (ICD-10 F10–F50 or F90–F98) and later risk of being diagnosed with any of the examined mental disorders. The years correspond to the time when the birth cohorts (1985–1997) were on the ninth grade (aged around 16). Model 1 was adjusted for sex and birth year. Model 2 was adjusted for sex, birth year, school class size, school's ninth grade size, area-level urbanicity, area-level morbidity, area-level education level, area-level employment rate, parental education, parental income, and parental mental health, with a random intercept per school.

© 2024 .Alho J et al. *JAMA Psychiatry*.
