# Supplementary material for: Transmission of Mental Disorders in Adolescent Peer Networks
Source: JAMA Psychiatry. 2024 May 22;81(9):882–8. doi: 10.1001/jamapsychiatry.2024.1126 (PMC11112494; doi:10.1001/jamapsychiatry.2024.1126)
Supplement: Supplement 2. — Data Sharing Statement [file jamapsychiatry-e241126-s002.pdf]

Data Sharing Statement

Alho. Transmission of Mental Disorders in Adolescent Peer Networks. *JAMA Psychiatry*. Published May 22, 2024. doi:10.1001/jamapsychiatry.2024.1126

Data
Data available: No

Additional Information
Explanation for why data not available: Data for the present study is property of Statistics Finland and Finnish Institute of Health and Welfare. The data are available from these authorities, but restrictions apply.
